# Supercritical Fluids and Nanoparticles in Cancer Therapy

**DOI:** 10.3390/mi13091449

**Published:** 2022-09-01

**Authors:** Iolanda De Marco

**Affiliations:** 1 Department of Industrial Engineering, University of Salerno, Via Giovanni Paolo II, 132, 84084 Fisciano, Salerno, Italy; idemarco@unisa.it; 2 Research Centre for Biomaterials BIONAM, University of Salerno, Via Giovanni Paolo II, 132, 84084 Fisciano, Salerno, Italy

**Keywords:** supercritical carbon dioxide, carrier-free nanoparticles, coprecipitated nanoparticles, in vitro and in vivo studies, anticancer effect

## Abstract

Nanoparticles are widely used in the pharmaceutical industry due to their high surface-to-volume ratio. Among the many techniques used to obtain nanoparticles, those based on supercritical fluids ensure reduced dimensions, narrow particle size distributions, and a very low or zero solvent residue in the powders. This review focuses on using supercritical carbon dioxide-based processes to obtain the nanoparticles of compounds used for the treatment or prevention of cancer. The scientific literature papers have been classified into two groups: nanoparticles consisting of a single active principle ingredient (API) and carrier/API nanopowders. Various supercritical carbon dioxide (scCO_2_) based techniques for obtaining the nanoparticles were considered, along with the operating conditions and advantages and disadvantages of each process.

## 1. Introduction

Fluids at supercritical conditions, i.e., at temperature and pressure values higher than those of the critical point, are used in different fields for various applications [1,2,3,4,5]. Their versatility is linked to the peculiarity of having some properties comparable to those of liquids (such as density) and others similar to those of gases (such as diffusivity). Among the various fluids used in the supercritical state, carbon dioxide is undoubtedly the most used. The reason for this choice is linked to its easily reachable critical values (P_c_ = 7.38 MPa and T_c_ = 31.1 °C), its non-toxicity, its relative cheapness, and the fact that at ambient conditions, it is in a gaseous state. Therefore, it separates without post-process treatments from liquids and the solids it comes into contact with during the process [6,7]. Techniques based on the use of supercritical carbon dioxide (scCO_2_) are increasingly used for the extraction of compounds with a high added value from solid matrices [8,9], to obtain microparticles [10,11] and nanoparticles [12,13], for the production of membranes [14,15], for the processing of liposomes [16,17], for the impregnation of active principle ingredients (APIs) into porous matrices [18,19], as a reagent during polymerization [20,21], and many others.

Although the advantages of scCO_2_-based processes are manifold, it should be noted that using high pressures causes relatively high production and operating costs for the plants. For this reason, scCO_2_-based procedures are used in high-added-value sectors, such as the pharmaceutical field. This sector is particularly interested in producing micro and nanoparticles because many drugs are poorly soluble in water and dissolve at meager rates in biological media. It is widely known that the size reduction improves the dissolution rate, ensuring a greater bioavailability of the API [22,23].

A category of drugs continuously studied and for which no expense is spared both in terms of the money invested in research and in terms of the purchase of materials is that of anticancer agents. Particles of nanometric and sub-micrometric dimensions are particularly interesting for cancer treatment. In order to understand how extensive the studies in this field are, Table 1 shows the most common types of cancer and some results obtained when nanoparticles are used.

The definition of nanoparticles is somewhat debated; we move from stringent definitions that consider nanoparticles with dimensions lower than 100 nm [37] to less restrictive ones in which the nanoscale is extended to particles smaller than 1–5 μm [38,39]. In the present paper, a compromise choice was made, and papers with powders with a diameter of less than 1 μm were considered.

Therefore, this review focuses on using supercritical fluids-based techniques to obtain nanoparticles (NPs) that can be used in cancer therapy. The different papers have been classified in the following sections and sub-sections: a) the production of carrier-free active principles; b) the coprecipitation of carrier and API nanoparticles.

## 2. Production of Nanoparticles

Supercritical fluids-based processes used for the production of NPs can be classified considering the role exerted by scCO_2_ with respect to the API to be micronized. In the majority of cases, scCO_2_ acts as the solvent, as the antisolvent, or as a co-solute. 

In the first group of processes, API is dissolved in scCO_2_ and precipitates because of a pressure drop that induces a rapid expansion; therefore, a highly diluted mixture consisting of solid particles and a gaseous phase is obtained [40]. This simple technique is commonly known as RESS (rapid expansion from supercritical solution) and has been used since the eighties [41]. The advantage of this technique is linked to the absence of organic solvents and surfactants for precipitation. At the same time, the cons are due to the low solubility of high molecular weight solids in scCO_2_. This disadvantage has been minimized by adding a solid cosolvent, which increases the solvating power of scCO_2_; in this case, the process is called RESS-SC (rapid expansion from supercritical solution with solid cosolvent) [42]. Other modifications of the conventional RESS process require the expansion of the supercritical solution to take place in a liquid receiving medium containing a surfactant in the so-called RESOLV (rapid expansion from supercritical solution into liquid solvents) [43] and RESSAS (rapid expansion from supercritical solution into aqueous solution) [44] processes. These processes are generally used to micronize pure compounds, which can be polymers [45] or active ingredients [46].

When supercritical CO_2_ is used as the antisolvent, the API is dissolved into an organic liquid miscible with scCO_2_ at the process conditions and precipitates because of the antisolvent effect of carbon dioxide on the solid. The process can be performed in batch or semi-continuous mode. In the first case, popularly known as GAS (gas antisolvent) [47], a batch constituted by the API and the organic solvent is loaded in a vessel in which scCO_2_ is pumped until the final pressure is obtained. In the semi-continuous process, the liquid and the scCO_2_ are continuously delivered into the precipitation vessel; the latter process is generally known as SAS (supercritical antisolvent) [48] or SEDS (solution enhanced dispersion by supercritical fluids) [49]. In some cases, an emulsion containing the API rather than a solution can be processed; in this case, the process can be called SEE (supercritical emulsion extraction) [50]. The main advantage of the antisolvent-based processes is related to the excellent control of the mean size and of the PSDs (particle size distributions) of the precipitated solid; conversely, high amounts of carbon dioxide have to be used to separate the residual solvent from the powders completely; moreover, water cannot be used as the solvent because, at the process conditions, scCO_2_ and water are not miscible. The latter limit, in some cases, has been overcome using a co-antisolvent that improves the mutual solubility of water and the antisolvent mixture [51].

In the third group of processes, scCO_2_ plays the role of the co-solute. In the SAA (supercritical assisted atomization) process [52], a controlled amount of scCO_2_ is solubilized in the API and solvent (which can be water or an organic solvent) liquid solution. Therefore, an expanded liquid is formed in a pre-chamber (a saturator) and is sprayed through a nozzle into an atmospheric pressure precipitation vessel. The pressure drop causes the precipitation of the solute; the solvent is evaporated thanks to a warm nitrogen stream fed to the precipitator [53]. The SAA process’s main advantage is the possibility of using both water and organic solvents to process the active principles. Conversely, the major drawback is that the SAA process temperature is higher than those employed in other supercritical carbon dioxide-based processes to assure solvent evaporation.

NPs used in the treatment and prevention of cancer can be formulated with or without the presence of a polymeric carrier. Carrier-free nanoparticles have generally been obtained using the RESS (or its derivatives) and SAS (or similar) process, whereas carrier and API NPs have been precipitated through SAS-type or SAA-type processes.

### 2.1. Carrier-Free Nanoparticles

Carrier-free nanodrugs have attracted increasing attention in cancer therapy because of their pharmacodynamics/pharmacokinetics, reduced systemic toxicity, and high drug loading capability [54,55]. The active principles processed through scCO_2_-based techniques are listed in Table 2, together with the method used to obtain the powders in the form of nanoparticles, the operating conditions employed, and the main results obtained.

**Figure 1:**
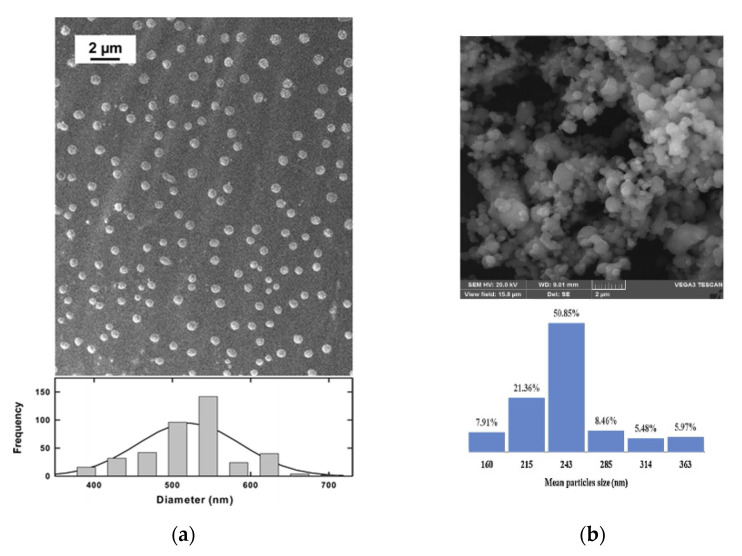
Exemplificative FESEM images and particle size distributions of nanoparticles obtained using scCO2. (**a**) Paclitaxel processed by the RESOLV process. Reprinted with permission from [57]. Copyright © 2022 American Chemical Society. (**b**) Capecitabine nanoparticles obtained by the GAS process. Adapted with permission from [61]. Copyright © 2022 Elsevier.

Using RESS-like and SAS-like techniques, particles in the nanometric range were obtained, as it is possible to observe from the results reported in Table 2. Therefore, the proposed techniques can be employed to obtain nanoparticles that can be used in chemotherapy when scCO_2_ is used as the solvent or antisolvent with respect to the active compound. Figure 1 shows exemplificative FESEM images and the corresponding particle size distributions.

Yao et al. [65] evaluated the in vitro and in vivo antitumor efficacy of camptothecin nanosuspension prepared by SAS and compared them with topotecan. The cytotoxicity of the two formulations was investigated against MCF-7 breast cancer, HCT-8 human ileocecal adenocarcinoma, and PC-3 human prostate cancer cell lines using an MTT assay; the dose-dependent toxicity in vivo during the treatment was investigated by body weight changes and relative organ weight variations. The SAS camptothecin nanosuspension presents about 6 times the in vitro cytotoxicity active against cell lines MCF-7, nearly the same in vivo antitumor activity, and lower toxicity than topotecan. Therefore, there are interesting expectations in the possible use of such suspensions to prepare non-toxic formulations with high antitumor efficacy.

Margulis et al. [70] prepared nanoparticles by solvent extraction from microemulsions in supercritical carbon dioxide. Celecoxib can promote blood vessel growth in cancer cell lines, but considering its hydrophobicity, it has to be administered in the form of nanoparticles to elicit a perceivable pharmacological response. Therefore, these authors prepared oil in a water microemulsion containing celecoxib (the active principle) and PLGA (poly lactide-co-glycolide) in the oily phase; the solvent extraction from this microemulsion by scCO_2_ yielded a solid powder composed of spherical nanoparticles, with an average size of 110 nm. The nanoparticles were dispersed in an injectable hydrogel constituted by PVA (polyvinylalcohol) and PVP (polyvinylpyrrolidone) cross-linked together. The resultant nanoparticles were administered subcutaneously to mice in a biocompatible hydrogel and caused a 4-fold increase in the blood vessel count in normally perfused skin compared with drug-free particles. 

In some cases, the efficacy of the NPs in treating the solid tumor has been tested by performing in vivo experiments. In these cases, an important parameter that has to be monitored is the tumor inhibitory rate (TIR), defined as:

$$TIR\;\%=\frac{tumor\;weight\;of\;control\;group-tumor\;weight\;of\;treated\;group}{tumor\;weight\;of\;control\;group}\times 100$$

**Figure 2:**
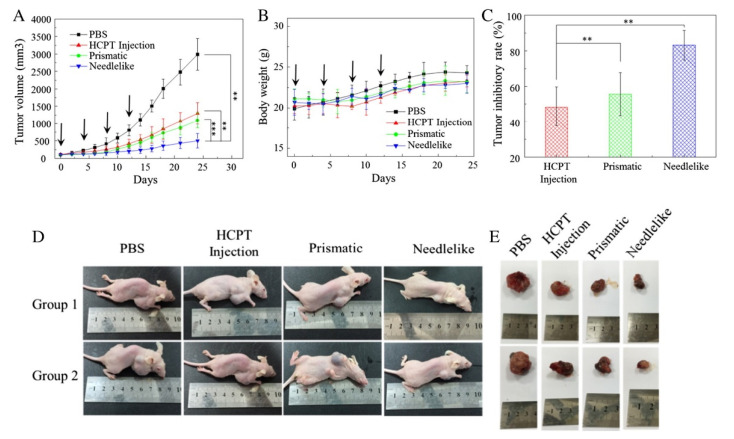
In vivo anticancer activity studies of 10-hydroxycamptothecin polymorphic nanoparticle dispersions. (**A**) Effects of tumor volume after intravenous injection of different dispersions. (**B**) Variation of the relative body weight of the mice after intravenous administrations. (**C**) The tumor inhibitory rate (TIR) after different treatments in A549 tumor-bearing nude mice. Data are represented as mean ± SD (n = 10). Statistical significance: ** *p* < 0.01; *** *p* < 0.005. (**D**) Representative tumor-bearing mice are treated with different formulations for 24 days. (**E**) Representative tumors collected at the end of the experiment. Reprinted with permission from [63]. Copyright © 2022 Elsevier.

For example, Wang et al. [63] used the SAS process to obtain a 10-hydroxycamptothecin polymorphic nanoparticle dispersion and compared the results in terms of tumor suppression using different polymorphic forms. Needle-shaped dispersions are promising candidates with a longer retention time in plasma, cellular internalization, tumor accumulation, and the effective suppression of tumor growth. In vivo anticancer activity results in mice in terms of the tumor volume, TIR, and dimensions of the tumors after the treatment are reported in Figure 2. In some cases, clinical trials have been carried out using powders obtained through SCF-based processes [71,72].

### 2.2. Coprecipitated Carrier and API Nanoparticles

As already mentioned before, the co-precipitation of a polymer and an API was obtained with techniques such as SAS or, in a fewer number of cases, SAA. The main results of the coprecipitation at nanometric dimensions are shown in Table 3.

**Figure 3:**
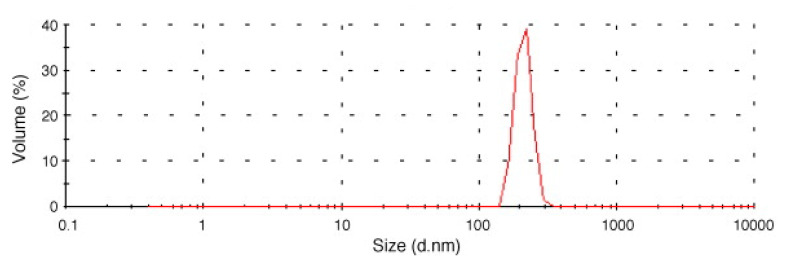
Particle size distribution of PCL and rosemary extract obtained by the SAS process. Reprinted with permission from [78]. Copyright © 2022 Elsevier.

Although the purpose of the SAS and SAA processes is often to obtain polymer and API coprecipitated microparticles [92,93], as evident from the results shown in Table 2, properly fixing the operating conditions, nanoparticles with a narrow size distribution have been obtained. For example, Figure 3 shows the particle size distribution of the nanoparticles constituted by polycaprolactone (PCL) and a rosemary extract [78].

**Figure 4:**
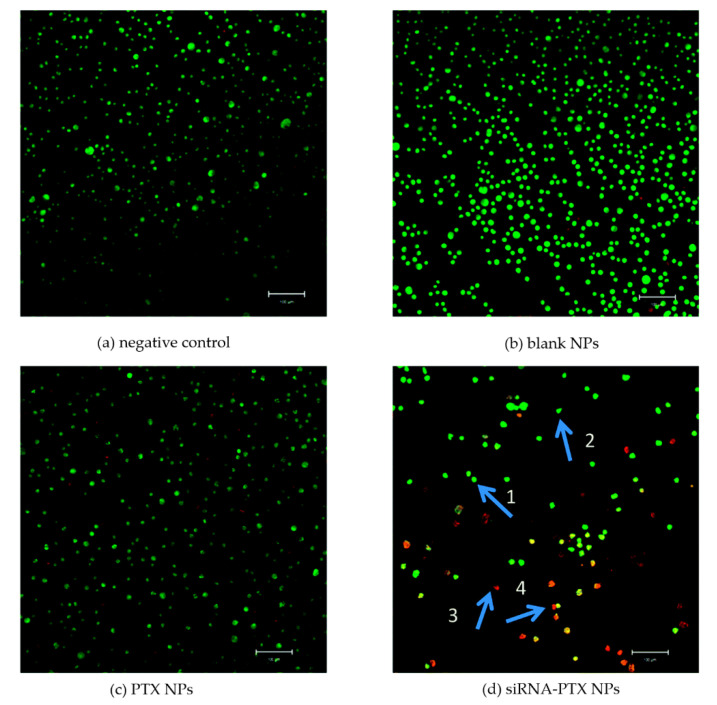
Antitumor activity of siRNA-paclitaxel NPs measured by AO/EB assay. Arrow 1 for the green round, arrow 2 for the green irregular, arrow 3 for the orange irregular, and arrow 4 for the orange round. Adapted with permission from [81]. Copyright © 2022 The Royal Society of Chemistry.

From the analysis of Table 3, it can be observed that in some cases, the authors have optimized the process operating parameters to obtain small and regular particles. In some papers, in vitro tests were also carried out using cancer cells; in others, in vivo experiments on mice have been performed. For example, Chen et al. [81] prepared, combining ionic gelation and SAS, composite particles containing siRNA and paclitaxel to be used for cancer chemotherapy to avoid drug resistance during the treatment. They used an acridine orange (AO)/ethidium bromide (EB) kit, which allows distinguishing through fluorescent microscopy the cells among normal (green round), viable apoptotic (green irregular), non-viable apoptotic (orange irregular), and dead (orange round). An evident antitumor effect of the siRNA and paclitaxel combined nanoparticles (revealed by the presence of many dead cells) can be observed in Figure 4.

Hua and Hua [72] obtained nanoparticles constituted by a camptothecin and bovine serum albumin-poly(methyl methacrylate) conjugate with an enhanced ability to kill cancer cells compared to the free drug in the solution both in vitro and in vivo. Indeed, the efficiency was demonstrated in vivo by injection into mice with subcutaneous colon cancer tumors. After 30 days, the size of the tumor treated with the coprecipitated NPs was unchanged, while that of the tumor treated with the unprocessed drug increased by 8 times.

**Figure 5:**
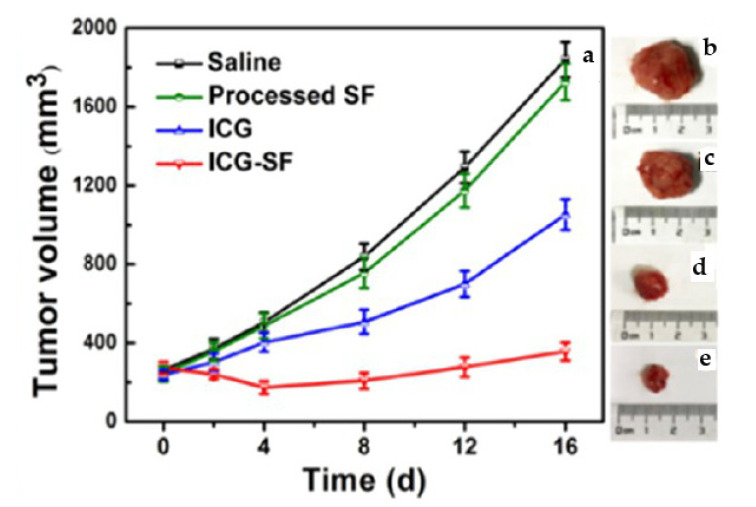
(**a**) Graphical representation of tumor volume vs. time in days after treatment of ICG-SF nanoparticles and corresponding pictures of excised tumors of mice treated with samples for 16 days, (**b**) saline, (**c**) SF nanoparticles, (**d**) ICG, and (**e**) ICG-SF nanoparticles. Reprinted with permission from [87]. Copyright © 2022, American Chemical Society.

In some cases, non-conventional chemotherapeutic strategies, such as photothermal therapy, are used [94,95]. Indeed, Chen et al. [87] prepared indocyanine green (ICG)—encapsulated silk fibroin (SF) nanoparticles for dual-triggered cancer therapy through the SAS process. The obtained coprecipitated powders showed excellent photothermal stability; the active principle was pH-responsive released from SF, specifically in the tumor acidic environment. In vitro and in vivo photothermal experiments have demonstrated that these ICG-SF nanoparticles strongly affect the tumor cells’ viability merely under light-induced hyperthermia. In Figure 5, the tumor volume reduction after treatment with ICF-SF NPs has been reported. After sixteen days, the tumor treated with the coprecipitated nanoparticles has a volume equal to less than half the volume of the tumor treated with the non-coprecipitated indocyanine green and less than a quarter of the volume of the tumor treated with the saline solution.

Supercritical carbon dioxide-based techniques have also been employed to prepare functionalized metal NPs for cancer theragnosis [96] and theranostics [97]. Indeed, A. S. Silva et al. [96] prepared gold nano-in-micro formulations for theragnosis and lung delivery. The engineered formulations present adequate morphology and flowability to reach the deep lung; moreover, the optimal biodegradation and release profiles enabled a sustained and controlled release of the embedded nanoparticles with enhanced cellular uptake. In other cases, systems that can deliver the anticancer drug to the site of action have been proposed. For example, M.C. Silva et al. [98] used scCO_2_-assisted spray drying to obtain nanocomposites constituted by strawberry-like gold-coated magnetite and ibuprofen that were charged in a chitosan matrix to treat lung cancer. 

Novel supercritical carbon dioxide-based techniques have also been proposed to obtain coprecipitated nanoparticles. For example, Zhang et al. [99] used a reverse emulsion-solution enhanced dispersion by supercritical fluids to precipitate 5-fluorouracil-loaded- PLLA–PEG/PEG nanoparticles. They obtained spherical NPs with smooth surfaces and a narrow particle size distribution. In vitro dissolution tests revealed a burst effect (18.4% of the drug released in the first hour), followed by a sustained release for about 120 h. Moreover, in vivo tests on mice demonstrated tumor growth inhibition (equal to 52% in the NPs and 46% in the case of the unprocessed 5-FU). These results suggest that 5-FU-NPs prepared using this supercritical carbon dioxide-based technique have potential anti-tumor applications as a controlled drug release dosage form.

## 3. Conclusions

This review focuses on using supercritical carbon dioxide-based techniques to produce nanoparticles useful in cancer treatment. Depending on the solubility of the active principle in carbon dioxide, it is possible to choose a process rather than another one. RESS-like methods are used when the active principle is soluble in carbon dioxide; they are used to process carrier-free drugs. In the SAS process (and similar ones), the carbon dioxide has the role of the antisolvent for the solute that has to be micronized; both carrier-free chemotherapic drugs and polymer and active ingredient coprecipitated powders have been successfully obtained at the nanoscale. The SAA process has been, instead, successfully applied to obtain coprecipitated nanoparticles. In vitro and in vivo tests have, in many cases, demonstrated the effectiveness of supercritical techniques in obtaining devices that can be used to treat tumors. The nanoparticles obtained with supercritical carbon dioxide-based methods are generally small and with small size distributions. Compared to other processes used in the preparation of nanoparticles, those based on SCF allow good control of the dimensions and distribution of the dimensions as the operating parameters vary. This represents a great advantage of these processes, which, therefore, become highly competitive with traditional ones in high-added value sectors such as the production of chemotherapy agents.

## Figures and Tables

**Table 1:** Summary of the use of nanoparticles to treat different cancers. NPs = nanoparticles; siRNA = short-interfering RNA.

Type of Cancer	Outcome	Reference
Bladder cancer	NPs used in in vitro cancer diagnostics, in vivo imaging enhancement, and drug loading techniques	[24]
Breast cancer	Three crucial biomarkers can be detected and accurately quantified in single tumour sections by the use of NPs conjugated with antibodies	[25]
Colorectal cancer	Effective release of cytotoxic drugs through targeted NPs; use of nanostructures with surface-bound ligands for the targeted delivery and ablation of the cancer	[26,27]
Kidney cancer	Efficacy of the cisplatin encapsulated into polybutylcyanoacrylate NPs	[28]
Lung cancer	Therapeutic and diagnostic systems at the nanoscale, which include polymeric NPs-based approaches, metal NPs-based approaches, and bio-NPs-based approaches	[29]
Lymphoma	Enhanced sensitivity and selectivity for earlier detection of circulating cancer biomarkers. In vivo, NPs enhance the therapeutic efficacy of anticancer agents, improving vectorization and delivery, and helping to overcome drug resistance	[30]
Melanoma	Anisamide-targeted NPs that can systemically deliver siRNA into the cytoplasm of B16F10 murine melanoma cells	[31]
Oral and oropharyngeal cancer	Polymeric, metallic, and lipid-based nanosystems that incorporate chemotherapeutics, natural compounds, siRNA, or other molecules	[32]
Pancreatic cancer	Gold NPs utilized for targeted drug delivery in pancreatic cancer leading to increased efficacy of traditional chemotherapeutics	[33]
Prostate cancer	Use of gold NPs to enhance radiation sensitivity and growth inhibition in radiation-resistant human prostate cancer cells	[34]
Thyroid cancer	Temperature-sensitive poly(N-isopropylacrylamide-acrylamide-allylamine)-coated iron oxide magnetic NPs as targeted drug carriers for treatments of advanced thyroid cancer	[35]
Uterine and ovarian cancer	Nanostructured probes are a new class of medical tool that can simultaneously provide imaging contrast, target tumor cells, and carry a wide range of medicines resulting in better diagnosis and therapeutic precision	[36]

**Table 2:** Carrier-free nanoparticles. 5-FU = 5-Fluorouracil; API = active principle ingredient; CC = cancer cells; CIS = cisplatin; CPC = capecitabine; CPT = Camptothecin; DCM = dichloromethane; DMSO = dimethylsulfoxide; EtOH = ethanol; GA = gambogic acid; GAS = gas antisolvent; md = mean diameter; HCPT = 10-hydroxycamptothecin; NPs = nanoparticles; P = pressure; PTX = paclitaxel; RESOLV = rapid expansion from supercritical solution into liquid solvents; RESS = rapid expansion from supercritical solution; RESS-SC = rapid expansion from supercritical solution with solid cosolvent; SAS = supercritical antisolvent; T = temperature.

Technique	API	Application	Operating Conditions	Main Results	Reference
scCO_2_ used as the solvent with respect to the API					
RESOLV	GA	lung, breast, and other CC	P = 25 MPa; T = 55 °C	md = 251.2 nm ± 85.6 nm; enhanced anticancer efficacy of the NPs compared to that of commercial GA	[56]
	PTX	ovarian, breast, lung, colon, head, leukemia, and neck CC	P = 31 MPa; T = 40 °C	md = 530 nm ± 85 nm; paclitaxel NPs are effective, with an antineoplastic activity comparable to that of the commercial paclitaxel formulation	[57]
RESS	CIS	head, neck, bladder, ovarian, and lung CC	P = 30 MPa; T = 40 °C	md = 200–300 nm; reduced toxic side effects in cancer patients undergoing CIS-based therapy	[58]
	Piroxicam	bladder, colon, prostate, and nonmelanoma skin CC	P = 20–35 MPa; T = 60–70 °C	md = 176 nm ± 53 nm; the results on different batches confirmed the robustness and reproducibility of the process	[59]
RESS-SC	Aprepidant	prevention of nausea and vomiting caused by chemotherapic drugs	P = 15–33 MPa; T = 35–65 °C	md = 23–523 nm; the dissolution rate coefficient was up to 8.2 times higher than that of the unprocessed API	[60]
scCO_2_ used as the antisolvent with respect to the API					
GAS	CPC	breast, colorectal, and gastric CC	P = 12–16 MPa; T = 35–55 °C	md = 243 nm at the optimal operating conditions; lower crystallinity of processed NPs leading to higher solubility and faster dissolution	[61]
SAS	5-FU	lung CC	P = 10–15 MPa; T = 40 °C	md = 248 nm using methanol-DCM 50:50 as the organic solvent; dry powder inhaler formulations with lactose as the carrier with an increase up to 21% of the respiratory fraction	[62]
	HCPT	broad spectrum of human CC	P = 10–14 MPa; T = 30–40 °C	md = 600 nm; shape- and polymorph-dependent tumor suppression observed in vitro and in vivo; the tumor inhibitory rate was 68% in needle-shape NPs	[63]
	CPT	broad spectrum of human CC	P = 10–25 MPa; T = 35 °C	md = 250 nm ± 20 nm; optimization of the micronization conditions and demonstration of no API degradation	[64]
	CPT	broad spectrum of human CC	P = 20 MPa; T = 35 °C	md = 251 nm ± 20 nm; with respect to topotecan, 6 times in vitro cytotoxicity activeagainst cell lines MCF-7, nearly the same in vivo antitumor activity with lower toxicity	[65]
	CPT	broad spectrum of human CC	P = 14 MPa; T = 40 °C	md = 390–870 nm depending on the organic solvent used; smaller particles obtained by using EtOH/DMSO because the solubility of CPT in the solution was lower	[66]
	Curcumin	colorectal CC	P = 22–22.5 MPa; T = 31–32.5 °C	md = 230–240 nm; enhanced anticancer effect on colorectal cancer cells (HCT116); reduced cytotoxicity on normal cells (NCM460) compared to curcumin-DMSO and 5-Fu	[67]
	Genistein	breast, ovarian, and prostate CC	P = 8.5–12 MPa; T = 40 °C	md = 254 nm; increased 24 h-plasma concentration by 2.6-fold after orally administrated to rats	[68]
	Taxol	ovarian, breast, and lung CC	P = 10–25 MPa; T = 35–68 °C	md = 150 nm; not induced degradation of the API	[69]

**Table 3:** Coprecipitated nanoparticles. 5-FU = 5-Fluorouracil; AO/EB = acridine orange/ethidium bromide; BSA = bovine serum albumin; CPT = Camptothecin; DXT = dextran; HCPT = 10-hydroxycamptothecin; API = active principle ingredient; CS = chitosan; DOX = doxorubicin hydrochloride; ELR = elastin-like recombinamer; FA-CS = folate-conjugated chitosan; FA-DEX = folate-conjugated dextran; FA-HSA = folate-conjugated human serum albumin; HP-β-CD = hydroxypropyl-β-cyclodextrin; ICG = indocyanine green; md = mean diameter; MTT = 3-[4,5-dimethylthiazole-2-yl]-2,5-diphenyltetrazolium bromide; PCL = polycaprolactone; PLGA = poly(lactide-co-glycolide); PLLA = poly(L-lactic acid); PLLA-PEG-PLLA = poly(L-lactide)–poly(ethylene glycol)–poly(L-lactide) triblock copolymer; PMMA = poly(methyl methacrylate); PTX = paclitaxel; PVP = polyvinylpyrrolidone; SAA = supercritical assisted atomization; SAS = supercritical antisolvent; SF = silk fibroin; siRNA = short-interfering RNA; T_m_ = temperature in the mixer; T_p_ = temperature in the precipitator.

Carrier	API	Operating Conditions	Main Results	Reference
scCO_2_ with the rule of the antisolvent				
HP-β-CD+PVP	PTX	P = 8.3 MPa; T = 40 °C	In vivo tests showed excellent antitumor activity in female mice bearing human mammary tumor xenografts; high degree of tumor growth inhibition with 100% complete tumor regressions and 80% tumor-free survivors	[73]
BSA-PMMA	CPT	P = 10 MPa; T = 40 °C	md = 310 nm ± 27 nm; in vitro and in vivo anticancer activity against human colorectal cancer cells	[72]
ELR	Docetaxel	P = 9.5–11 MPa T = 35 °C	md = 40 nm; increased water solubility up to fifty orders of magnitude; controlled API release profile; in vitro measurements demonstrated an enhanced effect of the API in breast cancer cells compared to healthy endothelial cells	[74]
FA-CS	HCPT	P= 20 MPa; T = 35 °C	Particle size in the range of 123–182 nm, suitable for intravenous Injection, and useful in the treatment of HCPT-sensitive tumors	[75]
FA-DEX	CPT	P = 10–20 MPa; T = 40–60 °C	md = 182 nm; attainment of tumor-targeted NPs	[76]
FA-HSA	HCPT	P= 25 MPa; T = 35 °C	HCPT NPs with a md = 119 nm and FA-HSA-HCPT NPs with a md = 234 nm; sustained release of the API and high affinity for tumor cells in vitro	[77]
PCL	Rosemary extract	P = 30 MPa; T = 40 °C	md = 255 nm; in vitro release exhibited an initial burst release within the first 15 min	[78]
PLGA	5-FU	P = 11 MPa; T = 36 °C	In vitro release profiles revealed a burst effect on the first day, followed by a sustained-release phase; an MTT assay performed on human lung carcinoma demonstrated the activity of the API when coprecipitated with PLGA	[79]
PLLA	DOX+ siRNA-CS	P = 8 MPa; T = 30 °C	Sustained release of DOX and higher anticancer efficacy in drug-resistant cells (human small cell lung cancer) than those treated with free DOX or DOX-PLLA	[80]
PLLA-PEG-PLLA	PTX+ siRNA-CS	P = 12 MPa; T = 35 °C	md = 323 nm; in vitro antitumor effect demonstrated by AO/EB assay; the drug-loaded NPs induced the apoptosis and death of cells	[81]
PLLA	PTX	P = 12 MPa; T = 33 °C	In vitro prolonged cytotoxicity against the proliferation of nonsmall-cell lung cancer A549 and ovarian cancer SKOV3 cell lines	[82]
PLLA	Tamoxifen	T = 13 MPa; T = 38 °C	Controlled delivery aimed at reducing the side effects of the API	[83]
PVP	β-Carotene	P = 8.5–10 MPa; T = 40 °C	md = 250 nm ± 50 nm; 10 times increase of the API dissolution rate	[84]
PVP	Folic acid	P = 9–15 MPa T = 35–40 °C	md in the range 50–810 nm depending on the process conditions; shorter dissolution time than the unprocessed API	[85]
PVP	Quercetin	P = 9 MPa T = 40 °C	md = 470 nm; dissolution rate from the coprecipitated NPs 10 times faster than the unprocessed API	[86]
PVP	Rutin	P = 9 MPa T = 40 °C	md = 840 nm; dissolution rate from the coprecipitated NPs 3.19 times faster than the unprocessed API	[86]
SF	ICG	P = 10 MPa T = 35 °C	In vitro and in vivo photothermal experiments showed that ICG-SF NPs were capable of devastating tumor cells under light-induced hyperthermia	[87]
SF	Curcumin	P = 20 MPa	md lower than 100 nm; improved inhibition effect against colon cancer cells	[88]
scCO_2_ with the rule of the co-solute				
CS	DOX	P = 8–12 MPa; T_m_ = 70 °C; T_p_ = 90 °C	md in the range 120–250 nm; in vitro drug release profiles showed a pH-responsive release with an initial burst; the SAA process did not alter the activity of DOX	[89]
CS	siRNA	P = 10 MPa; T_m_ = 70 °C; T_p_ = 90 °C	Controlled and sustained release of the API; in vivo biodistribution assessment of the powders in healthy mice showed deep lung diffusion	[90]
DXT	Curcumin	P = 9.2 MPa; T_m_ = 85 °C; T_p_ = 100 °C	md = 400 nm ± 130 nm; complete dissolution of the API in about 30 min	[91]
HP-β-CD	Curcumin	P = 9 MPa; T_m_ = 80 °C; T_p_ = 100 °C	md = 510 nm ± 160 nm; complete dissolution of the API in about 30 min	[91]
PVP	Curcumin	P = 8 MPa; T_m_ = 80 °C; T_p_ = 80 °C	md = 430 nm ± 140 nm; complete dissolution of the API in a reduced time (from a few minutes to 70 min depending on the PVP/API ratio) with respect to the unprocessed API	[91]

## Data Availability

Not applicable.
